# Implementation of Liquid Biopsy in Non-Small-Cell Lung Cancer: An Ontario Perspective

**DOI:** 10.3390/curroncol31100449

**Published:** 2024-10-08

**Authors:** Daniel Breadner, David M. Hwang, Don Husereau, Parneet Cheema, Sarah Doucette, Peter M. Ellis, Shaqil Kassam, Natasha Leighl, Donna E. Maziak, Shamini Selvarajah, Brandon S. Sheffield, Rosalyn A. Juergens

**Affiliations:** 1 Verspeeten Family Cancer Centre, London Health Sciences Center, London, ON N6A 5W9, Canada; 2 Department of Laboratory Medicine and Molecular Diagnostics, Sunnybrook Health Sciences Centre, Toronto, ON M4N 3M5, Canada; 3 School of Epidemiology and Public Health, University of Ottawa, Ottawa, ON K1N 6N5, Canada; 4 Division of Medical Oncology, William Osler Health System, Brampton, ON L6R 3J7, Canada; 5 Department of Medicine, University of Toronto, Toronto, ON M5S 1A1, Canada; 6 IMPACT Medicom Inc., Toronto, ON M6S 3K2, Canada; 7 Division of Medical Oncology, Juravinski Cancer Centre, Hamilton, ON L8V 5C2, Canadajuergensr@hhsc.ca (R.A.J.); 8 Department of Oncology, McMaster University, Hamilton, ON L8S 4L8, Canada; 9 Southlake Stronach Regional Cancer Centre, Newmarket, ON L3Y 2P9, Canada; 10 Division of Medical Oncology and Hematology, Princess Margaret Cancer Centre, University Health Network, Toronto, ON M5G 2C4, Canada; 11 Department of Thoracic Surgery, The Ottawa Hospital, Ottawa, ON K1Y 4E9, Canada; 12 Laboratory Medicine Program, Division of Genome Diagnostics, University Health Network, Toronto, ON M5G 2C4, Canada; 13 Department of Laboratory Medicine and Pathobiology, University of Toronto, Toronto, ON M5S 1A1, Canada; 14 Division of Advanced Diagnostics, William Osler Health System, Brampton, ON L6R 3J7, Canada

**Keywords:** liquid biopsy, non-small-cell lung cancer (NSCLC), molecular testing, targeted therapies, circulating tumour DNA (ctDNA)

## Abstract

Lung cancer is the leading cause of cancer-related deaths in Canada, with non-small-cell lung cancer (NSCLC) accounting for the majority of cases. Timely access to comprehensive molecular profiling is critical for selecting biomarker-matched targeted therapies, which lead to improved outcomes in advanced NSCLC. Tissue biopsy samples are the gold standard for molecular profiling; however, several challenges can prevent timely and complete molecular profiling from being performed, causing delays in treatment or suboptimal therapy selection. Liquid biopsy offers a minimally invasive method for molecular profiling by analyzing circulating tumour DNA (ctDNA) and RNA (cfRNA) in plasma, potentially overcoming these barriers. This paper discusses the outcomes of a multidisciplinary working group in Ontario, which proposed three eligibility criteria for liquid biopsy reimbursement: (1) insufficient tissue for complete testing or failed tissue biomarker testing; (2) suspected advanced NSCLC where tissue biopsy is not feasible; and (3) high-risk patients who may deteriorate before tissue results are available. The group also addressed considerations for assay selection, implementation, and economic impact. These discussions aim to inform reimbursement and implementation strategies for liquid biopsy in Ontario’s public healthcare system, recognizing the need for ongoing evaluation as technology and evidence evolve.

## 1. Introduction

In Canada, lung cancer accounts for the most diagnoses and deaths among all cancer types per year, placing a significant burden on Canadians and the healthcare system [1]. Non-small-cell lung cancer (NSCLC) is the most common type of lung cancer, accounting for approximately 88% of histologically defined cases in Canada [2]. Prognosis for NSCLC is poor with a 5-year survival rate below 20%, owing to its aggressive nature and large proportion of patients presenting with advanced or metastatic disease [2].

Despite this poor prognosis, biomarker-matched targeted therapy has demonstrated improved efficacy over non-targeted regimens in patients with advanced, molecularly defined NSCLC, contributing to an overall improvement in survival rates for NSCLC over the last few decades [3]. This personalized treatment approach currently relies on (1) timely comprehensive tissue molecular profiling of all currently known targetable genomic alterations (including alterations in *EGFR*, *ALK*, *ROS1*, *MET*, *ERBB2*, *BRAF*, *KRAS*, *RET*, and *NTRK* [4,5] and (2) access to biomarker-matched targeted therapy.

As of 1 June 2021, Ontario Health implemented the Comprehensive Cancer Biomarker Testing Program, which funds reflex tissue testing of all actionable genomic biomarkers by next-generation sequencing (NGS) in all patients with newly diagnosed NSCLC [6]. This highlights Ontario Health’s recognition of the evidence supporting the benefit of molecular profiling in NSCLC, in combination with personalized treatment options when available. All actionable biomarkers (except *ERRB2*) have matched targeted therapies approved by Health Canada [7], some of which are publicly funded in Ontario or may be accessed through clinical trials, compassionate use, self-pay, co-pay, or private insurance.

Despite widespread access to comprehensive tissue molecular profiling, several challenges prevent patients with advanced NSCLC from receiving biomarker-informed care. Approximately 5–16% of patients with NSCLC may not have adequate samples for comprehensive NGS due to the inability to perform a tissue biopsy as a result of patient comorbidities, a tissue biopsy procedural failure, or insufficient sample quality or quantity for testing [8,9]. Additionally, patients with rapidly progressing disease may require treatment prior to the availability of tissue testing results, leading to biomarker-uninformed treatment decisions associated with suboptimal clinical outcomes [10,11].

Comprehensive molecular profiling of circulating tumour DNA (ctDNA) and RNA (cfRNA) in plasma by NGS (herein referred to as “liquid biopsy testing”) is a minimally invasive method for evaluating actionable genomic biomarkers, potentially overcoming some of the challenges posed by tissue testing alone. Although not a substitute for histologic diagnosis of lung cancer, liquid biopsy testing can complement tissue testing in the diagnostic pathway through faster sample acquisition and processing compared with using tumour tissue, as well as the inclusion of genetic material from multiple sites that might reflect tumour heterogeneity more adequately [12]. International oncology associations have endorsed the use of liquid biopsy testing in advanced stage, treatment-naive NSCLC [4,13,14,15]. However, routine liquid biopsy testing in Ontario is still in the early steps of implementation as a standard-of-care test, with it being only available through private funding, research protocols, or institutional funding at select centres, leaving many patients without access.

Given the challenges of tissue molecular profiling and the importance of genotyping results for appropriate front-line treatment decisions, there is a significant need for public funding of liquid biopsy for Ontario patients with NSCLC to ensure equitable and timely access to molecular profiling. A multidisciplinary group of specialists from across Ontario was formed to discuss strategies for the integration of liquid biopsy into the comprehensive molecular profiling of NSCLC tumours and to consider which strategies should be reimbursed in a public healthcare system. This paper summarizes these discussions, which include a proposal of eligibility criteria for reimbursement, as well as considerations for assay selection, implementation, and economic analysis. It is intended to aid in decisions on public reimbursement and implementation of liquid biopsy testing for advanced lung cancer patients in Ontario but is not a formal consensus or evidence-based guideline. While the remit of the working group was to focus specifically on Ontario (a province of 14 million), this work may also provide lessons for other publicly funded healthcare systems.

## 2. Methodology

A multidisciplinary working group was formed to propose criteria for public reimbursement of plasma ctDNA testing for Ontario patients with advanced lung cancer. Participants included Ontario specialists in the diagnosis and management of lung cancer (six medical oncologists, two pathologists, one clinical molecular geneticist, and one thoracic surgeon) as well as one health economist. Three virtual meetings were organized to discuss (1) eligibility criteria for reimbursement of liquid biopsy testing; (2) test requirements and selection considerations; and (3) implementation and economic considerations. The content for each meeting was prepared and facilitated by four core advisors (D.B., D.M.H., R.A.J., and D.H.) with assistance from a medical writer (S.D.). A summary of meeting discussions was shared with participants for further commentary and used to develop this report.

## 3. Discussion on Eligibility Criteria

The working group proposed three distinct eligibility criteria for public funding of liquid biopsy testing in patients with advanced NSCLC. Table 1 presents eligibility criteria prioritized based on unmet needs and strength of evidence. These criteria are aligned with recommendations from international guidelines, as well as recommendations from the National Institute of Excellence in Health and Social Services in Quebec (INESSS; Table 1) [4,13,14,15,16].

### 3.1. Eligibility Criterion 1: Patients with Advanced NSCLC Where Tissue Is Insufficient for Complete Testing or Tissue Biomarker Testing Failed

The advisors agreed that liquid biopsy testing for patients with advanced NSCLC where tissue is insufficient for complete biomarker testing or tissue biomarker testing failed represents the highest-need group with the strongest supporting evidence. In prospective studies comparing molecular profiling by tissue and liquid biopsies, rates of insufficient tissue for complete genotyping of actionable biomarkers ranged from 11 to 50% and liquid biopsy testing was able to identify actionable alterations in 10 to 37% of these patients [17,18,19,20,21,22,23,24,25].

The sensitivity of liquid biopsy testing for detecting actionable genomic alterations in reference to tissue testing ranges from 63 to 81% [17,20,21,22,23,26,27,28,29,30,31,32,33,34,35,36,37]. Sensitivity varies by testing method and alteration type, with reported sensitivity rates frequently below 60% for the detection of novel fusions and copy-number variations. The sensitivity of liquid biopsy testing is also significantly impacted by low tumour burden, composition of blood vessels in and around the lesion, and decreased ctDNA shedding rates in some tumours, leading to potentially false-negative results [38,39]. Although methods for more accurately identifying true-negative results are under investigation, currently, a negative result on plasma testing should be considered uninformative and prompt a tissue re-biopsy and testing or repeating the blood draw [13]. Despite this, liquid biopsy is an important complement to tissue re-biopsy given the potential for significantly faster turnaround time (range of 7 to 10 days total and 6 to 27 days faster from sample receipt to result reporting compared with tissue biopsy testing) [17,20,21,36,37]. The minimally invasive nature of liquid biopsy also improves patient comfort and circumvents complications associated with tissue re-biopsy.

Although targeted therapies in NSCLC have been approved based on detection of genomic alterations in tissue, there is substantial evidence that supports similar clinical outcomes in patients selected for matched targeted therapy via liquid biopsy [18,19,21,25,26,35,40,41,42,43,44,45,46,47,48,49]. Studies also indicate that lower allele frequencies, which are typically reported with liquid biopsy testing, do not impact the observed efficacy of targeted agents [40,43,46,47]. The international guidelines suggest that the high analytical and clinical specificity of liquid biopsy testing increases confidence that a positive liquid biopsy result can reliably guide treatment decisions [13].

### 3.2. Eligibility Criterion 2: Patients with Suspected Advanced Lung Cancer for Whom a Tissue Biopsy Is Not Feasible (e.g., Due to Bone-Only Disease, Inaccessible Primary Tumours, or Poor Lung Function) and with a Clear Indication for Treatment

Patients with suspected advanced NSCLC who are unable to undergo a tissue biopsy due to technical limitations or comorbidities cannot receive a histological diagnosis, which presents a significant clinical challenge. Although rates vary by study population, approximately 3% of patients with suspected advanced NSCLC are unable to receive a histological diagnosis due to the inability to receive a tissue biopsy or a tissue biopsy failure [17,50]. Liquid biopsy provides an opportunity to match these patients to targeted therapy, where management options would otherwise be limited.

Evidence for the utility of liquid biopsy in patients with suspected advanced lung cancer where a tissue biopsy is not feasible is somewhat limited. A single clinical trial and several case series, including an example from an Ontario-based community hospital, have demonstrated success in using liquid biopsy in this setting, particularly to match patients to EGFR tyrosine kinase inhibitors [51,52,53,54]. In addition, results from studies evaluating a liquid-biopsy-first strategy in patients with suspected advanced NSCLC in order to accelerate the time to treatment may be extrapolated to support the feasibility and clinical utility of liquid biopsy in suspected advanced NSCLC [17,55,56]. For example, in the ACCELARATE study conducted at the Princess Margaret Hospital in Toronto, Ontario, 70% of patients with suspected advanced NSCLC had biopsy-proven advanced NSCLC [17]. Among those with confirmed non-squamous NSCLC (60%), 23% started targeted therapy before their tissue NGS results were available and 12% had actionable alterations only detected through liquid biopsy testing, supporting the benefit of a liquid-biopsy-first strategy. Interestingly, among the patients without a histological diagnosis of NSCLC, three of five patients who did not undergo biopsy and all three patients with insufficient tissue from biopsy for diagnosis had tumour-associated variants detected in their plasma. Additionally, 3 of 18 patients diagnosed with other malignancies had informative liquid biopsy results. Given the substantial percentage of patients diagnosed with small-cell lung cancer or other malignancies, the authors concluded that liquid biopsy remains a complementary rather than an alternative approach to tissue biopsy, with histological confirmation still needed to guide treatment. While the working group agrees that tissue confirmation should be prioritized, in cases where it is not feasible, these data suggest that liquid biopsy has the potential to inform diagnoses and/or treatment decisions. This sentiment is reflected in guidelines from the European Society for Medical Oncology (ESMO) [13]. Additionally, other Canadian provinces, including Quebec, New Brunswick, and Nova Scotia, are planning to implement liquid biopsy testing for patients who are unable to undergo a tissue biopsy [16,57,58].

The working group acknowledges the limitations of liquid biopsy when NSCLC is not histologically confirmed, including the possibility of false-negative or false-positive results. However, given that these patients have no alternative for diagnosis and molecular profiling, it was felt that the potential benefits for liquid biopsy in the case where an actionable genomic alteration is identified and targeted therapy could be administered would outweigh these risks.

### 3.3. Eligibility Criterion 3: Patients with Suspected or Confirmed Advanced NSCLC with a High Risk of Deterioration or Death before Tissue Results Are Expected to Be Reported (e.g., within 14–21 Days)

The third criterion for liquid biopsy consideration is for those patients with a high risk of deterioration before tissue profiling results could be reported (e.g., <14–21 calendar days). The timeliness in reporting NGS results to inform therapeutic decisions is critical in a rapidly aggressive disease like NSCLC, where a 3–4 week delay in treatment may be associated with a 10–13% mortality rate, and many patients become too sick to consider initiating therapy [59]. Overall, over one-third of patients with advanced NSCLC will die within the first 2 months of diagnosis [60]. There is a particularly high need in Ontario for fast comprehensive molecular profiling, as turnaround times for tissue testing often far exceed recommended thresholds [61]. However, as tissue testing remains the gold standard for molecular profiling, liquid biopsy testing should be considered as a complementary strategy for expediting molecular profiling results in select patients rather than a widespread solution to long turnaround times with tissue profiling. Therefore, the panel encourages centres to adopt quality assessment and improvement programs to ensure reporting for tissue profiling meets recommended timelines.

Examples of patients that may fall under this third criterion include hospitalized patients where expedited molecular profiling results can “rescue” patients through initiation of targeted therapy or guide referral to palliative care, in the scenario where no feasible treatment is identified, both of which could reduce time in hospital. Success of this strategy has been demonstrated in single-centre studies where the use of liquid biopsy in patients with suspected metastatic NSCLC helped to diagnose and inform management [51,62].

## 4. Discussion on Test Selection and Requirements

A pan-cancer NGS panel consisting of key cancer-associated genes focused on actionability was deemed most suitable for adoption in routine practice. This is justified given the expectation that liquid biopsy will be used across several tumour types, as is recommended in the recent ESMO ctDNA guidelines [13]. Although a pan-cancer NGS panel may have an initial increased cost compared with tumour-specific panels, the incremental cost difference is expected to be low and will reduce cost and time related to the validation of multiple panel tests. A pan-cancer NGS panel also has the advantage of utility in patients with clinical suspicion of carcinoma but without a known diagnosis.

The pan-cancer NGS panel selected should, at a minimum, cover all actionable genomic biomarkers in NSCLC as recommended by Cancer Care Ontario [5], as well as other relevant biomarkers that may inform management decisions (e.g., *TP53* alterations) and relevant genomic biomarkers for other tumour sites (e.g., ESR1 and AKT pathway genes for breast cancer). Commercial tests offered through private laboratories in the United States, such as FoundationOne^®^ Liquid CDx (Foundation Medicine Inc., Boston, MA, USA) or Guardant 360^®^ CDx (Guardant Health, Palto Alto, CA, USA) exceed these requirements and are available in Ontario through patient-pay at a high cost. Some commercial, off-the-shelf panel tests that can be performed in hospital laboratories also meet these requirements [63,64,65]. These assays differ by panel size, as well as enrichment chemistry, which impact test specifications and performance in certain scenarios [66]. For example, compared with hybrid-capture methods, amplicon-based methods have the advantage of a relatively faster turnaround time, lower cost, a more streamlined workflow, better sensitivity for single-nucleotide variants and insertions/deletions, and the ability to accommodate lower nucleic acid input. In contrast, hybrid-capture-based panels have more flexibility in increasing scale or sequencing depth, produce less background noise due to their ability to capture specific target regions, have a higher sensitivity for copy-number changes (such as deletions in the *BRCA1* and *BRCA2* genes in prostate and ovarian cancer patients), and often utilize ctDNA rather than cfRNA for the detection of fusions, rendering them potentially more sensitive for fusion detection with currently available technology.

Rather than adopting a commercial panel test, some laboratories may opt for a custom panel. This allows for more flexibility in selection of gene targets as new evidence emerges and allows for flexibility in reagent selection, which can decrease the cost per test. However, custom panels typically require a larger upfront investment in terms of validation cost and require a bioinformatician to build a custom analysis pipeline. In contrast, commercial panels may be more costly per test but have a shorter validation process and more streamlined analytical pipelines to offer. Thus, the validation process and costs, as well as data analysis requirements and bioinformatics resources available at each testing site, will impact test selection. Compatibility with sequencing systems already used in testing laboratories and the sample capacity of different assays/systems should also be considered. The ideal sample capacity aims to maximize the number of samples that can be processed simultaneously while minimizing delays caused by waiting for a sufficient running capacity to be reached. The panel recommends that centres looking to implement liquid biopsy testing should carefully consider these factors when selecting an assay for validation, understanding each assay’s strengths and limitations. As current public funding for tissue-based molecular profiling in Ontario is delivered as a flat reimbursement price per test, the selection of a liquid biopsy test will also depend on the funding price per test set by Ontario Health. Decisions on funding criteria and cost allocation are discussed in Section 6 below.

An important challenge with all liquid biopsy testing methods is the identification of mutations related to clonal haematopoiesis (CH). CH occurs when somatic mutations arise in haematopoietic stem cells, leading to a clonal expansion of mutated blood cells. It is prevalent in the general population and is related to ageing [67], although patients with solid tumours tend to exhibit higher rates of CH as well [68]. As genomic alterations associated with CH can overlap with driver mutations found in tumour DNA, CH variants identified on plasma cell-free DNA testing can be erroneously classified as tumour-derived alterations, leading to inappropriate selection of therapy [67]. Mutations in the *KRAS* gene have been identified in CH, which, in the context of liquid biopsy analysis in NSCLC, could prevent further genotyping efforts based on mutual exclusivity with other driver genes, potentially leading to missed detections of actionable alterations [69,70].

CH and tumour variants can be differentiated through simultaneous sequencing of peripheral blood mononuclear cells (PBMCs), which would significantly increase the cost of testing. Education on CH for pathologists and medical oncologists is needed to better understand when CH subtraction via PBMC sequencing is required and to ensure potential CH variants are appropriately reported and interpreted. Bioinformatics programs are being explored using machine learning to filter out CH variants based on distinct biophysical and genomic features such as the fragment size of the cell-free DNA, without the need for additional sequencing [67,71]. This can be performed at a low cost by analyzing data from the existing assay, thus negating the need to perform concurrent sequencing of PBMCs. Such programs should be considered in the data analysis workflow as they become available.

## 5. Discussion on Implementation Considerations

Considerations for the implementation of liquid biopsy for patients with advanced NSCLC were discussed with a focus on the following topics: testing delivery model, factors along the workflow that may impact performance, expectations on turnaround time, and additional resources to aid in implementation.

Testing delivery for liquid biopsy could be structured in several ways, including through in-house testing at each treatment centre, centralized testing at larger academic hospitals, or outsourcing of testing to private laboratories, following a similar funding model to non-invasive pre-natal testing in Ontario for high-risk pregnancies. Currently, nine centres perform tissue molecular profiling for the province of Ontario. This is a mixed delivery model, mostly involving centralization of testing at large academic centres, with a few community centres offering in-house testing specifically for their patients. A similar mixed model could be used for the delivery of liquid biopsy molecular profiling; however, the total number of central testing centres and catchment areas may differ from the current tissue testing structure. This will be influenced by the type of technology and NGS assay adopted by each laboratory and the sample volumes each centre is expected to accommodate within a reasonable cost and timeframe.

**Figure 1:**
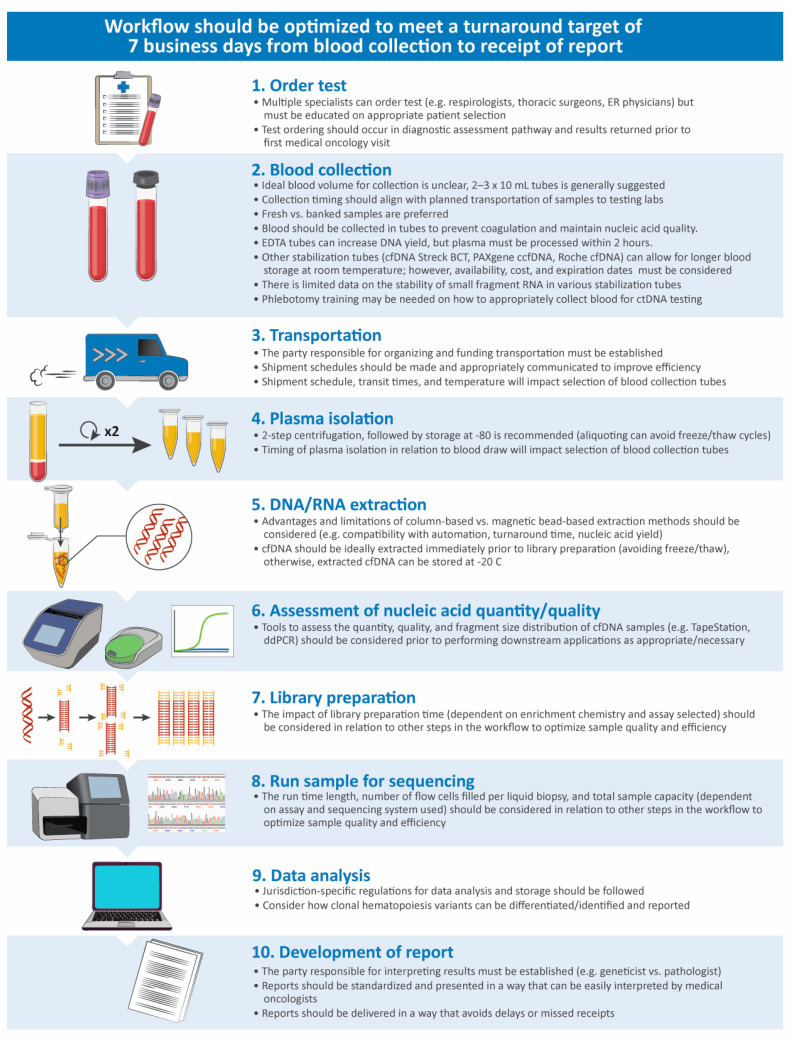
Considerations for optimization along the liquid biopsy testing workflow [74,75,76]. cfDNA, cell-free DNA; ctDNA, circulating tumour DNA; ddPCR, digital drop polymerase chain reaction; EDTA, ethylenediaminetetraacetic acid; ER, emergency room.

Factors impacting the performance and timing of liquid biopsy testing across the workflow are presented in Figure 1. Generally, the establishment of a structured pathway and good communication between sites and specialists across the entire workflow is needed to deliver high-quality, fast results. As turnaround time is paramount for reaping the benefits of liquid biopsy testing, formal targets for turnaround time should be set. While international guidelines have not made clear recommendations for liquid biopsy testing turnaround times, clinical studies report a turnaround of 7–10 business days from the receipt of samples to the generation of reports. Although a complete consensus was not reached, advisors agreed that turnaround times for liquid biopsy molecular profiling should not exceed guideline-recommended turnaround times for tissue testing (10 business days from the receipt of samples in molecular testing labs to reporting) [72,73]. A maximum turnaround time of 7 business days from sample receipt to reporting was suggested as feasible in most centres; however, a faster turnaround time of 5 business days would be optimal and is essential for patients at a high risk of deterioration before tissue testing can be completed (criterion 3). For these targets to be consistently met across all testing centres, funds should be allocated for formal monitoring of turnaround times and to acquire additional pathology and technologist resources and training where needed.

Validation remains a major barrier to the adoption of liquid biopsy testing for lung cancer patients in Ontario in terms of costs, time, and sample acquisition. A support program that can connect laboratory leads from different sites, allowing them to share knowledge and testing samples, would help to overcome this barrier.

## 6. Discussion on Economic Considerations

The implementation of universal access to liquid biopsy in Ontario through public reimbursement requires Ontario Health to review estimates of cost effectiveness and total budget impact for liquid biopsy across various scenarios and cost models to determine appropriate eligibility criteria and cost per test. The panel discussed three studies modelling the economic impact of liquid biopsy testing in NSCLC from a Canadian healthcare perspective [8,9,77]. The analytic techniques, modelling parameters, and outcomes measured differed in each study; however, all studies reported an increase in life-years with the use of liquid biopsy testing versus standard-of-care tissue testing only. In a study by Ezeife et al., which modelled the cost effectiveness of liquid biopsy added to standard-of-care testing versus tissue testing alone in patients with advanced non-squamous NSCLC and a ≤10 pack-year smoking history, an incremental cost savings of CAD 3065 per patient and a gain of 0.02 quality-adjusted life-years were reported over a 2-year horizon [77]. This was driven largely by the increase in actionable targets identified, leading to higher use of more effective targeted therapy compared to more costly chemo-immunotherapy. Studies by Patel et al. and Johnston et al. estimated a 3-year budget impact for the use of liquid biopsy testing for patients with NSCLC and insufficient tissue for standard-of-care tissue testing [8,9]. Budget impacts of CAD 14.7 million and CAD 4.4 million over 3 years were reported, which was largely dependent on modelling inputs for the rate of insufficient tissue (16% and 5%, respectively). Of note, these budget impact studies included costs for commercial, out-of-country tests in their models, at a price of >CAD 6000, which may not be feasible for public reimbursement in Ontario.

The panel also discussed some factors that may impact patient outcomes and costs that should be included in health economic models but are often not appropriately captured. For example, the faster turnaround time for liquid biopsy testing could prevent delays in treatment initiation or uninformed first-line treatment selection while awaiting tissue molecular profiling results, both of which are associated with poorer outcomes in NSCLC [10,11,59]. In a real-world study of patients with lung cancer in a community-based Ontario hospital, a median turnaround time of 36.5 days (interquartile range: 29.5–47 days) was reported for tissue testing results (*EGFR* and *ALK*), with only 20% of patients having biomarker results at the first consultation [61]. In this study, 8% of patients were prescribed chemotherapy, 16% were prescribed best supportive care, 20% were hospitalized, and 7% died while awaiting biomarker results [61], some of which could have been avoided with access to liquid biopsy testing.

## 7. Summary

Universal access to liquid biopsy testing for patients with advanced NSCLC remains a significant unmet need in Ontario. Our working group proposed key eligibility criteria that should be prioritized for public funding of liquid biopsy testing in Ontario based on the highest clinical needs and the robustness of the supporting evidence (Table 1). While these recommendations have been made for the Ontario publicly funded healthcare system, they may also provide insights into appropriate options for other public healthcare systems worldwide. However, specific recommendations by the working group for jurisdictions outside of Ontario are beyond the scope of this work.

Our proposed eligibility criteria are aligned with recommendations for the reimbursement of liquid biopsy testing from Quebec’s health technology assessment agency, INESSS, with the exception of a fourth criterion described by INESSS to fund liquid biopsy for patients with NSCLC progressing on targeted therapy, where the identification of resistance mutations could lead to the use of a listed second-line therapeutic agent [16]. Our working group did not include this population in our discussions as it was felt that the use of liquid biopsy for this purpose was still in the research phase, except in the few patients who may have received first-generation EGFR tyrosine kinase inhibitors and developed an *EGFR T790M* resistance mutation. However, this emphasizes that although reimbursement of liquid biopsy for patients meeting our proposed criteria is an important initial step, the role of liquid biopsy in molecular profiling is expected to evolve as technological advancements and new evidence emerge, which will necessitate continuous reassessment and adaptation of funding and eligibility criteria.

As another example, while current research suggests adopting a liquid-biopsy-first model for molecular profiling early in the lung cancer diagnostic pathway has the potential to improve patient outcomes by accelerating clinical decision making and treatment initiation [17,55,56], current technological constraints and high costs limit its feasibility within a publicly funded healthcare system. However, as the costs associated with liquid biopsy testing decrease and the technology matures, this model could become a viable option.

**Figure 2:**
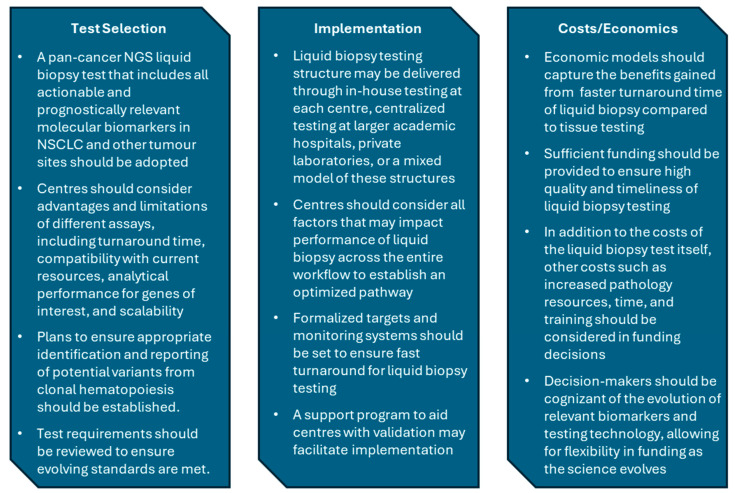
Key messages from workgroup discussions on of test selection, implementation, and cost analysis/economics. NGS, next-generation sequencing; NSCLC, non-small-cell lung cancer.

In our working group meetings, considerations for assay selection, test delivery, and cost analysis were discussed (Figure 2). As with eligibility criteria, panel requirements for liquid biopsy tests will evolve and require a reassessment to ensure all biomarkers informing treatment selection in NSCLC are included. Given the aggressive nature of NSCLC and its potential for rapid deterioration, delivery of liquid biopsy molecular profiling in a way that prioritizes turnaround time will be critical to give patients with advanced NSCLC the best chance to access potentially life-extending targeted therapy. The establishment of target metrics and a process for monitoring turnaround time will help to ensure that the quality of care is consistent, regardless of where a patient lives. Since liquid biopsy is rapidly accessible, even for patients living far from diagnostic centres, the implementation of a liquid biopsy testing program may also improve equity in lung cancer care across the province.

Molecular profiling via liquid biopsy is a high-complexity test that requires more intensive data analysis and interpretation to avoid the false negatives and positives uniquely associated with this analyte. As such, a successful liquid biopsy testing program will not only rely on investment in high-quality assays and technology but also on investment in resources such as pathologist/clinical laboratory geneticists’ and technologists’ time, acquisition, and training. These additional costs should be factored into reimbursement decisions.

Lastly, as liquid biopsy serves as an adjunct to tissue biopsy in the care pathway of patients with advanced NSCLC, timely turnaround for molecular profiling on tumour samples is still an important issue. Thus, quality improvement programs addressing turnaround times beyond the recommended targets for tissue molecular profiling should also be pursued.

## 8. Conclusions

There is substantial evidence supporting the benefit of liquid biopsy for patients with advanced NSCLC and a critical unmet need for universal access to this diagnostic test across Ontario through public reimbursement. Beyond molecular profiling in advanced NSCLC, there is a role for liquid biopsy in other tumour sites, as well as emerging applications in early disease stages [13,39]. Liquid biopsies that utilize other fluids, such as urine, saliva, or cerebrospinal fluid, as well as other analytes, such as circulating tumour cells, various cfRNA species (e.g., miRNA), exosomes, metabolites, and microbial cell-free DNA, are also emerging as tools that inform the management of patients with cancer [78]. Thus, building expertise and infrastructure for liquid biopsy testing now may ensure accelerated adoption when additional applications are ready to be implemented in routine care. We hope that this initiative has laid a foundation for ongoing discussions around the implementation of liquid biopsy in Ontario for advanced NSCLC and beyond.

## Figures and Tables

**Table 1:** Proposed eligibility criteria for liquid biopsy testing in advanced NSCLC and alignment with international recommendations.

Eligibility Criteria for Liquid Biopsy Testing *	Alignment with International Guidelines				
	ESMO 2022 [13]	NCCN 2024 [4]	IASLC 2021 [14]	ASCO PCO 2022 [15]	INESSS 2024 [16]
Criterion 1: Patients with advanced NSCLC where tissue is insufficient for complete testing or tissue biomarker testing failed	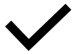	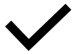	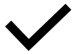	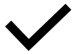	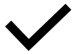
Criterion 2 ^†^: Patients with suspected advanced lung cancer for whom a tissue biopsy is not feasible (e.g., due to bone-only disease, inaccessible primary tumours, or poor lung function) and with a clear indication for treatment	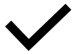	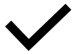	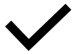	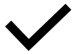	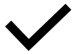
Criterion 3: Patients with a suspected or confirmed advanced NSCLC with a high risk of deterioration or death before tissue results are expected to be reported (e.g., within 14–21 days)	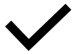	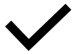	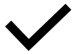	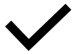	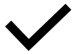

* Criteria are prioritized based on unmet needs and the strength of supporting evidence. ^†^ Guidelines support the use of a liquid biopsy when a tissue biopsy is not feasible and/or the use of a liquid biopsy prior to diagnosis or at the time of the diagnostic biopsy; however, its use in suspected advanced NSCLC is not consistently stated. A tissue diagnosis is still strongly recommended where feasible. ASCO PCO, American Society of Clinical Oncology Provisional Clinical Opinion; NSCLC, non-small-cell lung cancer; ESMO, European Society for Medical Oncology; IASLC, International Association for the Study of Lung Cancer; INESSS, Institut National d‘Excellence en Santé et en Services Sociaux (National Institute of Excellence in Health and Social Services); NCCN, National Comprehensive Cancer Network.

## Data Availability

No new data were created or analyzed in this study. Data sharing is not applicable to this article.
